# Overview of Primary and Secondary Metabolites of *Rugulopteryx okamurae* Seaweed: Assessing Bioactivity, Scalability, and Molecular Mechanisms

**DOI:** 10.3390/md23090351

**Published:** 2025-08-30

**Authors:** Ana Minerva García-Cervantes, José A. M. Prates, José Luis Guil-Guerrero

**Affiliations:** 1 Food Technology Division, ceiA3, CIAMBITAL, University of Almería, 04120 Almería, Spain; agc874@ual.es; 2 CIISA—Centro de Investigação Interdisciplinar em Sanidade Animal, Faculdade de Medicina Veterinária, Universidade de Lisboa, 1300-477 Lisboa, Portugal; 3 Associate Laboratory for Animal and Veterinary Sciences (AL4AnimalS), 1300-477 Lisboa, Portugal

**Keywords:** *Rugulopteryx okamurae*, alginate, diterpenoids, polyphenols, antioxidant activity, Invasive species valorisation

## Abstract

*Rugulopteryx okamurae* is an invasive brown alga that has colonised Mediterranean and northeastern Atlantic coastlines, posing significant ecological and economic challenges. Its biomass is rich in structurally diverse metabolites—including polysaccharides (alginate, fucoidan, laminaran), phlorotannins, diterpenoids, fatty acids, and peptides—many of which exhibit notable antioxidant, anti-inflammatory, antimicrobial, and anticancer activities. Comparative assessment of extraction yields, structural features, and bioactivity data highlights phlorotannins and diterpenoids as particularly promising, demonstrating low-micromolar potencies and favourable predicted interactions with key inflammatory and apoptotic targets. Algal polysaccharides exhibit various bioactivities but hold strong potential for scalable and sustainable industrial applications. Emerging compound classes such as fatty acids and peptides display niche bioactivities; however, their structural diversity and mechanisms of action remain insufficiently explored. Insights from in vitro and in silico studies suggest that phlorotannins may modulate NF-*κ*B and MAPK signalling pathways, while diterpenoids are implicated in the induction of mitochondrial apoptosis. Despite these findings, inconsistent extraction methodologies and a lack of in vivo pharmacokinetic and efficacy data limit translational potential. To overcome these limitations, standardized extraction protocols, detailed structure–activity relationship (SAR) and pharmacokinetic studies, and robust in vivo models are urgently needed. Bioactivity-guided valorisation strategies, aligned with ecological management, could transform *R. okamurae* biomass into a sustainable source for functional foods, cosmetics, and pharmaceuticals applications.

## 1. Introduction

Marine algae are recognised as a rich and diverse source of bioactive compounds with promising applications in the pharmaceutical, nutraceutical, and cosmetic industries. These include polysaccharides, polyphenols, flavonoids, carotenoids, proteins, peptides, and vitamins, all of which exhibit a broad spectrum of biological activities [1,2,3].

Polysaccharides such as fucoidan, laminarin, and carrageenan are abundant in marine algae and are known for their immunostimulatory, antiviral, anticancer, antioxidant, antibacterial, antifungal, anti-inflammatory, and anticoagulant properties [1,2]. Polyphenols, particularly phlorotannins from brown algae, display potent antioxidant, anticancer, antiviral, anti-diabetic, anti-allergic, antibacterial, antihypertensive, and immunomodulatory effects [1,3]. Similarly, carotenoids, such as fucoxanthin, exhibit antioxidant, anti-inflammatory, and antineoplasic activities [1,3].

Marine algae also provide bioactive proteins and peptides with demonstrated antimicrobial, antioxidant, and anticancer effects, making them attractive candidates for nutraceutical applications [1,3]. In addition, they are a valuable source of essential vitamins and minerals that enhance their nutritional quality and promote overall health [1,2].

These bioactives support a range of applications across industries. In pharmaceuticals, compounds derived from marine algae are being explored for their therapeutic potential against cancer, neurodegenerative diseases, diabetes, cardiovascular disorders, and infections [1,3]. In the food sector, algae are incorporated into functional foods and nutraceuticals due to their antioxidant, anti-inflammatory, and antimicrobial properties [1,3]. In cosmetics, their moisturising, anti-ageing, and skin protective effects have led to a growing interest in algae-based skincare formulations [4,5].

In recent decades, marine brown algae of the genus *Rugulopteryx* have attracted considerable attention due to their remarkable abundance of structurally diverse secondary metabolites. These include sulphated polysaccharides, phlorotannins, and unique diterpenoids, which have been shown to possess significant antioxidant, anti-inflammatory, and antimicrobial properties [6,7,8]. Nevertheless, despite the expanding diversity of these compounds, it remains unclear which chemical classes offer the most favorable balance of potency, selectivity, and scalability for pharmaceutical or nutraceutical applications. Furthermore, the precise molecular mechanisms through which these compounds exert their bioactivities are not yet fully elucidated. In this review, we therefore address two interrelated aims. Firstly, a systematic comparison of extraction yields, structural features, and in vitro efficacies across major metabolite families is conducted in order to identify those with the greatest translational promise. Secondly, we integrate structure–activity relationship data and mechanistic studies, drawing on insights from related brown algal genera, to elucidate how key metabolites modulate cellular pathways. This process highlights the most critical gaps for future in vivo validation and optimisation.

### 1.1. Biology and Ecology of Rugulopteryx okamurae

**Figure 1:**
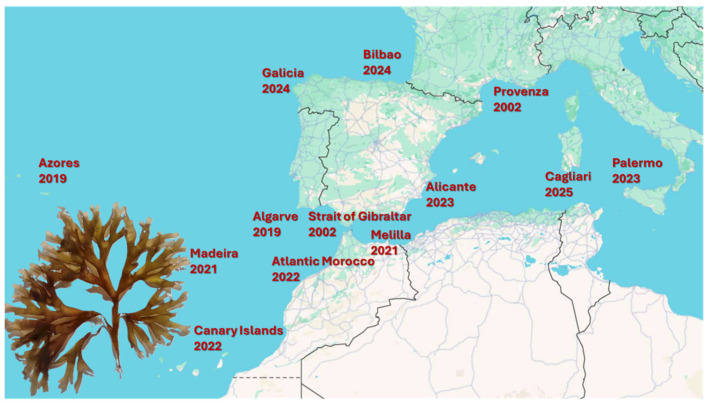
Coastlines that *R. okamurae* has colonised so far, along with the year of its first detection.

*R. okamurae* is a brown macroalga that is native to the northwestern Pacific Ocean [9,10,11]. First detected in the Strait of Gibraltar in 2015, it has since spread rapidly across the Mediterranean and north-eastern Atlantic. Figure 1 illustrates the European and African coasts that *R. okamurae* has colonised so far, along with the year of its first detection in each area.

This species exhibits a high degree of adaptability to diverse environmental conditions, including a broad thermal tolerance ranging from 10 to 30 °C, which facilitates its extensive colonisation. Furthermore, it possesses a high nitrogen storage capacity, thereby enabling growth in nutrient-rich waters [12,13].

In terms of nutrition, *R. okamurae* contains high levels of carbohydrates, fats, and ash, with a lower protein content. While it is a source of essential nutrients and trace elements, it has also been observed to accumulate toxic heavy metals. Extracts from *R. okamurae* have been shown to contain high levels of polyphenols and to exhibit antioxidant properties [9,10].

*R. okamurae* has been observed to colonise both the subtidal and intertidal zones, with a recorded depth of up to 50 m [9,10,14]. This species frequently becomes dominant, displacing native flora and fauna and reducing biodiversity. This alteration in turn impacts the structure and function of the ecosystem [9,14,15]. In areas subject to substantial invasion, there is a decline in fish diversity concomitant with an increase in the prevalence of opportunistic species [15]. Its chemical defences contribute to its resistance against herbivory and environmental pressures [16].

The management strategy encompasses the implementation of continuous monitoring through the utilisation of remote sensing techniques and the operation of sentinel stations [10,14]. The purple sea urchin (*Paracentrotus lividus*) has demonstrated potential as a biocontrol agent in laboratory studies [17]. Moreover, research is underway to explore the utilisation of its biomass in the production of biogas, compost, bioplastics, and pharmaceuticals [16,18].

Despite the considerable economic costs associated with the accumulation and removal of its biomass [14,16], the species also presents commercial opportunities through the extraction of valuable bioactive compounds [16].

In summary, *R. okamurae* is a highly invasive and resilient species, which has major ecological and economic consequences. To mitigate the impact of this phenomenon, there is a necessity for the implementation of integrated control strategies and sustainable biomass utilisation. Further research is needed to deepen our understanding of the organism’s biology and to inform the development of effective solutions [10,11,12,13,14,16,17].

### 1.2. Invasion Dynamics and Environmental Impact

*R. okamurae* has proliferated rapidly along the southern European coastline, particularly in the Mediterranean and north-eastern Atlantic regions, causing substantial ecological and socio-economic impacts [16,19]. Its expansion has been particularly notable along the Andalusian coast in southern Spain, where it has colonised submerged habitats at depths ranging from 0 to 50 m [20].

The ecological implications of this invasion are substantial. *R. okamurae* has displaced native species and altered the structure of benthic marine communities, resulting in a significant loss of natural variability and biodiversity [10]. In some areas, it has attained complete dominance, covering up to 100% of the substrate and supporting dense, yet uniform, epifaunal communities [13]. Its capacity for resilience to repeated marine heatwaves serves to enhance its invasive success, thereby raising concerns regarding the long-term functioning and stability of ecosystems [9].

In addition to the ecological damage resulting from the invasion, considerable economic losses have been incurred. In Tarifa, Spain, for instance, the financial burden of biomass removal and the disruption to marine activities is estimated to exceed three million euros on an annual basis, exerting a substantial impact on the fishing sector and public services [21].

**Figure 2:**
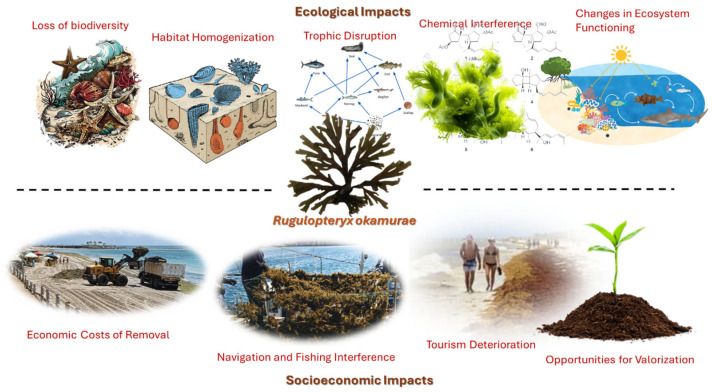
Socioeconomic and ecological impacts of *R. okamurae*.

The socioeconomic and ecological impacts of *R. okamurae* are illustrated in Figure 2.

The necessity of coordinated management strategies is therefore paramount in order to mitigate the aforementioned impacts. These measures must encompass localised removal, early detection, and long-term monitoring of its propagation. International collaboration is of pivotal significance in supporting the development of effective mitigation strategies, adapting public policies, and assessing the potential for biotechnological applications of biomass. However, most of the research has been concentrated on the Strait of Gibraltar and its adjacent waters, resulting in a paucity of data regarding the impacts in other vulnerable regions. These findings underscore the highly invasive behaviour of *R. okamurae* and highlight the necessity for further research into its spatial distribution, ecological impacts, and potential applications. Furthermore, the challenges associated with cross-border coastal management are revealed. Consequently, implementing a comprehensive monitoring program at a regional or basin-wide scale is essential to track distribution shifts and ecological impacts, providing the data needed for informed decision-making [22].

The invasion of *R. okamurae* poses significant ecological, economic, and policy challenges. Its aggressive propagation and adaptability have led to severe impacts on marine ecosystems and coastal economies. Although some valorisation options have been explored, there is an urgent need for more comprehensive research to deepen our understanding of *R. okamurae*’s biology, ecological interactions, and control mechanisms. Effective management will require sustained international collaboration and a multidisciplinary approach that combines impact mitigation with the development of sustainable utilisation strategies.

Some reviews on *R. okamurae* have been published, addressing its potential applications in biogas production, composting, bioplastics, and pharmaceuticals, with emphasis on the bioactive properties of its biomass [23]; management strategies based on sustainable valorization to promote marine ecosystem restoration and biotechnology, highlighting key bioactive compounds [16]; and economic assessments in Tarifa, Spain, underscoring the importance of prevention, early detection, and rapid response to mitigate the costs of future marine-coastal biological invasions [21]. Nevertheless, a comprehensive review specifically dedicated to the detailed chemical composition of *R. okamurae* and its associated bioactivities remains absent from the literature.

This review aims to address this gap.

## 2. Methodology

### 2.1. Focus Question

The composition in primary and secondary metabolites of *R. okamurae*, for assessing bioactivity, scalability, and molecular mechanisms.

### 2.2. Search Strategy

**Figure 3:**
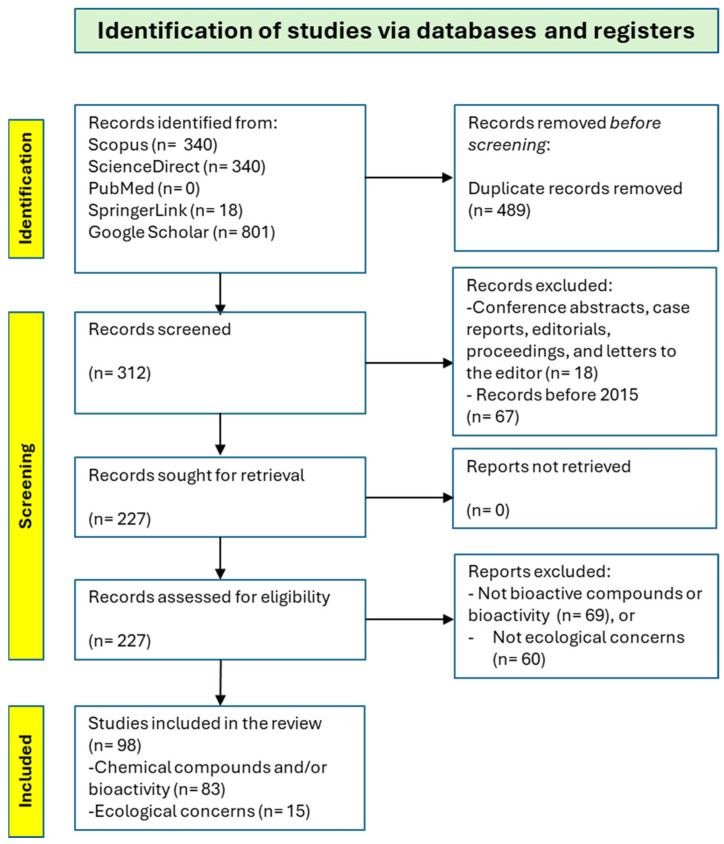
The research PRISMA flow diagram.

To ensure comprehensive coverage of the bioactive compounds, their bioactivities, and the structure–function relationships of *R. okamurae*, an extensive literature search was conducted using the main academic databases. The following bibliographic databases are to be consulted: PubMed, Scopus, ScienceDirect, SpringerLink, and Google Scholar. A targeted keyword strategy was applied, combining terms related to this alga and its biocompounds. Boolean operators (AND, OR) were employed to refine the searches—for example, “alginate AND anticoagulant” or “fatty acids OR lipids AND *Rugulopteryx okamurae*”. Furthermore, a process of backwards citation tracking of key reviews and primary studies was performed to identify relevant articles not captured through database queries (Figure 3).

### 2.3. Eligibility Criteria

The eligibility criteria employed in this systematic review were established by considering the research questions formulated according to the PICO (population, intervention, comparator, outcome) framework.

- Population: Species Rugulopteryx okamurae
- Intervention: Chemical composition and bioactivity
- Comparison: Positive effects vs. negative actions
- Outcome: Rugulopteryx okamurae, bioactive compounds, and bioactivity

The PICO framework is a widely utilised tool in the domain of systematic reviews, employed to formulate structured search strategies that ensure comprehensive and unbiased literature retrieval. It is a fundamental instrument within the paradigm of evidence-based practice, particularly in the domain of evidence-based medicine, used to formulate focused clinical or healthcare-related inquiries and the direction of the search for pertinent evidence. In this study, the PICO format was applied to define the inclusion criteria and to guide the selection of articles across multiple databases.

### 2.4. Inclusion Criteria

Inclusion was limited exclusively to peer-reviewed articles published in English that focused on marine-derived bioactive compounds from *R. okamurae* and reported at least one of the following: detailed structural characterization, bioactivity assays, biomedical or nutritional applications, or ecological implications. Although no strict date limitation was imposed, priority was given to research publications from 2015 to 2025, to capture the period during which this alga began colonizing European and African coastal regions. Foundational studies from earlier periods were selectively included when they provided essential context on bioactive compounds. A comprehensive synthesis of quantitative trends was achieved through meta-analyses and systematic reviews. Additionally, life-cycle assessments and sustainability reports were reviewed to offer insights into environmentally friendly extraction methods and scalability.

## 3. Overview of Primary and Secondary Metabolites

Macroalgae synthesise a diverse range of bioactive compounds, broadly categorised into primary and secondary metabolites. Primary metabolites are essential for the organism’s growth, development, and reproduction. These include carbohydrates, which provide energy and structural support; proteins, fundamental for cellular structure and function; lipids, vital for membrane integrity and energy storage; and nucleic acids, which store genetic information and regulate protein synthesis.

In contrast, secondary metabolites are not directly involved in basic metabolic processes but play crucial roles in ecological interactions such as defence, communication, and adaptation to environmental stressors. Among the most studied are flavonoids, valued for their antioxidant activity [24,25]; terpenoids, recognized for their antimicrobial and anti-inflammatory properties [25,26]; and alkaloids, which often exhibit potent antimicrobial effects [25,27]. Other notable groups include phenolic compounds, known for their antioxidant potential [24,26], and steroids, which contribute to anti-inflammatory [24,26].

Extraction and analysis of these compounds typically involve solvents such as methanol, chloroform, and ethyl acetate [24,26,27]. Analytical techniques like gas chromatography-mass spectrometry (GC-MS) and nuclear magnetic resonance (NMR) spectroscopy are frequently used to identify and characterise their structures [28,29].

Secondary metabolites from macroalgae exhibit a wide range of biological activities. Many possess antimicrobial properties effective against diverse pathogens [24,25,30], while flavonoids and phenolic compounds are especially noted for their strong antioxidant properties [24,28]. Additionally, several metabolites show promising anti-inflammatory activities [31].

These bioactivities make secondary metabolites highly valuable for various industrial applications. In the pharmaceutical sector, they are actively investigated for drug development and therapeutic use [30,31]. In cosmetics, their antioxidant and anti-inflammatory properties are harnessed in skincare formulations [25]. Within the food industry, certain compounds function as natural preservatives owing to their antimicrobial effects [25].

Although specific data on *R. okamurae* remain limited, broader insights into macroalgal metabolites provide a useful foundation for future research. These compounds play essential ecological roles and hold significant potential for application in health, food, and cosmetics. Targeted studies on *R. okamurae* are needed to elucidate its unique chemical profile and assess its industrial potential.

### 3.1. Polysaccharides

The polysaccharides of *R. okamurae* are primarily composed of holocellulose, containing significant amounts of glucose and glucuronic acid. Alongside essential nutrients and trace minerals, these polysaccharides contribute to the bioactive potential of *R. okamurae*, particularly in antioxidant applications. Other seaweeds provide a variety of polysaccharides with diverse biological activities, underscoring their broad potential across the food, pharmaceutical, and biomaterials industries [32,33,34,35]. Polysaccharides derived from brown algae—including *R. okamurae*—such as alginate and fucoidan are particularly notable for their diverse biological activities and potential applications.

A study investigated the potential of *R. okamurae* as a raw material to produce fermentable sugars, which can be converted into high-value-added products. The dietary fibre composition of this macroalga was analysed and compared with that of other brown and red macroalgae, revealing one of the highest contents of dietary fibre (27.3%) and cellulose (13.6%). These findings suggest that enzymatic hydrolysis of *R. okamurae* biomass could yield hydrolysates with a high concentration of reducing sugars [36]. Furthermore, *R. okamurae* biomass was subjected to hydrothermal and hydrothermal acid pretreatments, after which the pretreated material underwent enzymatic hydrolysis. This process yielded a hydrolysate with a reducing sugar concentration of nearly 25 g/L—an increase of 49.2% compared to non-pretreated biomass [37]. The extraction of polysaccharides from *R. okamurae* employs various techniques tailored to maximise yield while preserving compound integrity. One such technique is Microwave-Assisted Extraction (MAE), which uses pressurised hot water at 180 °C for 10 min to solubilise over 40% of the initial biomass. This technique yields an alginate recovery of 3.2%, phenolic content of 2.3%, and significant antioxidant activity attributed to the extracted polyphenols [32,38].

Ethanol extraction at a biomass-to-solvent ratio of 1:20 (*w*/*v*), combined with either homogenization or overnight agitation, has yielded higher extract recoveries compared with aqueous extraction metho. This makes it an efficient alternative for isolating phenolic compounds and other bioactives [39]. Additionally, solid-state fermentation (SSF) with *Aspergillus awamori*, followed by enzymatic hydrolysis, significantly increases the production of total reducing sugars—primarily glucose—highlighting the potential of biological pre-treatment strategies to enhance extraction efficiency [40].

Following extraction, various purification methods are employed to refine the isolated compounds. Considering algal biomasses, dialysis is typically applied to purify polysaccharides by removing low-molecular-weight impurities, resulting in preparations enriched with high carbohydrate and sulphate content [41].

Structural characterisation of the extracts is essential to confirm the identity and purity of the compounds. Nuclear Magnetic Resonance (NMR) and Fourier-Transform Infrared (FTIR) spectroscopy are used to validate the purity and structural integrity of sodium alginate extracted from *R. okamurae*. FTIR spectra reveal characteristic peaks associated with uronic acids, rhamnose, and sulphate groups [8]. High-performance liquid chromatography coupled with tandem mass spectrometry (HPLC-MS/MS) identifies key polyphenols, such as gallic acid and chlorogenic acid, that contribute to antioxidant activity [32]. Furthermore, electron microscopy provides high-resolution images to confirm the morphological characteristics of the extracted compounds [8].

#### 3.1.1. Alginates

Alginate is a hydrophilic, anionic polysaccharide predominantly extracted from brown seaweed. Its structure comprises linear chains of *β*-d-mannuronic acid (M) and *α*-l-guluronic acid (G), linked by 1→4 glycosidic bonds. These monomers are arranged in three distinct block patterns: homopolymeric mannuronate (MMM) and guluronate (GGG) regions, as well as alternating sequences (MGMG). The composition and arrangement of these blocks affect alginate’s flexibility, solubility, and gel-forming capacity, especially the G-blocks, which form strong gels through ionic cross-linking with divalent cations, such as calcium (Ca^2+^), barium (Ba^2+^) and strontium (Sr^2+^) via the “egg-box” model [42]. These properties make alginate highly valuable across various industrial and biomedical applications.

Recent studies have demonstrated successful extraction of alginate from *R. okamurae*, with its gelation performance in the presence of Ca^2+^ ions comparable to that of commercial alginates. This highlights its potential as a sustainable and functional alternative [8,42,43].

The various methods to extract alginates from brow seaweeds are detailed in Table 1.

Two main primary methods have been employed to extract alginate from *R. okamurae* biomass: conventional chemical extraction [8,43] and microwave-assisted hydrothermal extraction [38]. Both approaches are effective; the microwave-assisted method offers advantages in speed and environmental sustainability, while the conventional method produces high-purity alginate with excellent gel-forming properties.

One of alginate’s most distinctive features is its gel-forming ability, which underpins its extensive application in tissue engineering and drug delivery systems. Its biocompatibility, non-toxicity, and mild gelation conditions make it especially suitable for encapsulating cells, therapeutic agents, and other bioactive compounds [8,44,45]. These characteristics highlight alginate’s importance in medical and pharmaceutical fields and emphasize its potential to add value to biomass derived from invasive species such as *R. okamurae*.

**Figure 4:**
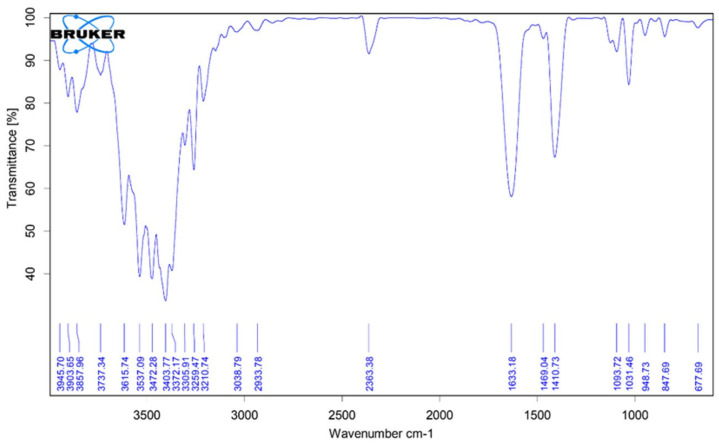
FTIR spectrum of sodium alginate purified from *R. okamurae* [8].

The FTIR spectrum of purified alginate extracted from *R. okamurae* displays the characteristic absorption bands typical of sodium alginate (Figure 4). When compared to commercial sodium alginate (product no. 180947-500 G, Sigma-Aldrich, Madrid, Spain), the following key signals can be assigned: O-H stretching at 3403.77 cm^−1^; symmetric and asymmetric COO^−^ stretching at 1633.18 and 1410.73 cm^−1^, respectively; and C-O-C stretching vibrations at 1093.72 and 1031.46 cm^−1^, corresponding to *β*-D-mannuronic acid (M) and *α*-L-guluronic acid (G) residues. Additional peaks include a band at 847.69 cm^−1^, attributed to the C_1_-H deformation of M residues, and a signal at 948.73 cm^−1^, associated with C-O stretching of uronic acid residues. Notably, the region between 1230 and 1280 cm^−1^, typically associated with S=O stretching vibration in sulphated polysaccharides like fucoidan, shows no significant absorption. This absence confirms the high purity of the alginate extracted from *R. okamurae* [8].

**Figure 5:**
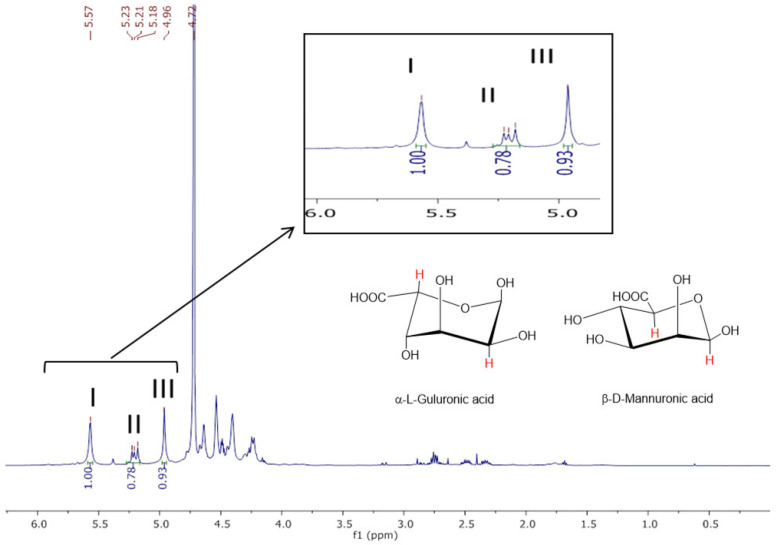
^1^H-NMR spectrum of sodium alginate purified from *R. okamurae*. Signal I: guluronic acid anomeric proton (HG1); signal II: overlap of mannuronic acid anomeric proton (HM1) and H5 of alternating blocks (HGM5); signal III: guluronic acid H5 proton in homopolymeric G blocks [8].

The ^1^H-NMR spectrum of sodium alginate and its interpretation are shown in Figure 5.

**Figure 6:**
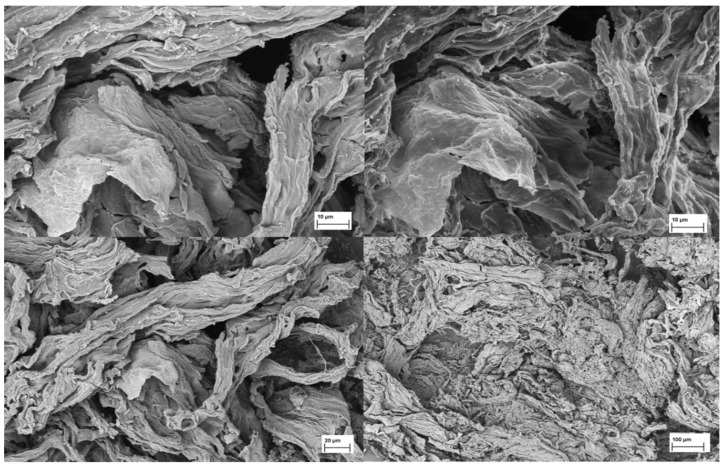
Images of purified and lyophilised sodium alginate from *R. okamurae* obtained with a Field Emission Scanning Electron Microscope at different scales [8].

Several images of purified and lyophilised sodium alginate from *R. okamurae* obtained with a Field Emission Scanning Electron Microscope at different scales are displayed in Figure 6.

#### 3.1.2. Fucoidans

It is supposed that *R. okamurae*, as other representatives of brown algae, should contain fucoidan, but there is no structural investigation of this polysaccharide at present. Fucoidan is a sulphated polysaccharide primarily found in the cell walls of brown algae. Its backbone is primarily composed of *α*-L-fucopyranose units, typically linked via (1→3) or alternating (1→3) and (1→4) glycosidic bonds [46,47]. The fucose residues are frequently sulfated at the C-2 and/or C-4 positions, imparting a strong negative charge that is critical to fucoidan’s biological bioactivity. In addition to fucose, fucoidan may contain minor amounts of other monosaccharides such as galactose, mannose, glucose, xylose, and uronic acids. Its precise composition and linkage pattern vary depending on the algal species and the extraction method [48,49].

The structure of fucoidan is highly heterogeneous, exhibiting considerable variation in molecular weight, degree and position of sulfation, monosaccharide composition, and degree of branching. While this structural diversity is a defining characteristic, it also presents challenges in establishing precise structure-function relationships [47,50]. Nonetheless, this complexity underpins the broad range of biological activities attributed to fucoidan.

Fucoidan is well known for its anticoagulant, antiviral, antitumor, antioxidant, and immunomodulatory properties [47,51]. These bioactivities have generated significant interest in their potential applications across pharmaceutical, nutraceutical, and biomedical sectors. However, the variability in structure among fucoidans—depending on algal source and extraction conditions—can lead to significant differences in bioactivity. This highlights the need for standardised extraction protocols and comprehensive structural characterisation in future studies.

#### 3.1.3. Laminarans

It is presumed that *R. okamurae*, in line with other brown algae, contains laminaran; nevertheless, structural elucidation of this polysaccharide remains unavailable. Laminarans are *β*-d-glucans with a backbone primarily composed of (1→3)-linked *β*-d-glucopyranose residues, with some (1→6) linkages and terminal mannitol [46,48]. The ratio of (1→3) to (1→6) linkages can vary, influencing their biological activity [48]. These polysaccharides have been noted for their potential antitumor activities and ability to inhibit colony formation in cancer cells [48].

#### 3.1.4. Comparison Among Polysaccharides of Brown Algae

A summary of the polysaccharides identified in brown algae, all of which are presumed to occur in *R. okamurae*, is presented in Table 2.

Alginate is a linear copolymer composed of *β*-d-mannuronic acid and *α*-l-guluronic acid residues arranged in block wise sequences. Its well-established gel-forming ability makes it particularly valuable in biomedical applications, including tissue engineering and controlled drug delivery. The ratio and arrangement of mannuronate and guluronate residues play a crucial role in determining alginate’s mechanical strength and rheological behavior [42,44,45].

Fucoidan is a sulfated polysaccharide primarily composed of *α*-l-fucose units, typically linked through alternating (1→3) and (1→4) glycosidic bonds. Its high structural variability—shaped by species, extraction method, and environmental conditions—contributes to a wide spectrum of biological activities. Fucoidan is particularly noted for its anticoagulant, antiviral, and antitumor properties, which have been extensively explored for therapeutic applications [46,47,51].

Laminaran, a storage *β*-glucan found in brown algae, consists of a (1→3)-linked glucose backbone with occasional (1→6) branches. It has demonstrated anticancer potential, particularly in inhibiting the formation of cancer cell colonies, and is also recognized as a dietary fibre with immunomodulatory effects [46,48].

Collectively, these polysaccharides play a central role in the functional and therapeutic potential of brown algae, supporting a wide range of applications in biomedicine, nutrition, and biotechnology.

#### 3.1.5. Applications of Polysaccharides Extracted from *R. okamurae*

Polysaccharides extracted from *R. okamurae* demonstrate promising applications across multiple fields. One notable use is in the development of biofertilizers, where these compounds enhance plant growth and contribute to bioremediation efforts, supporting sustainable agriculture practices [8]. Additionally, the bioactive polysaccharides exhibit antioxidant and anti-inflammatory properties, highlighting their potential in pharmacological applications for health promotion and disease prevention [39].

Beyond agriculture and medicine, the nutritional profile *R. okamurae* supports its potential role in the food and biotechnology industries. Its bioactive compounds may be utilised as functional ingredients, enhancing both the nutritional value and health benefits of food products [52].

The extraction, purification, and structural characterisation of these compounds involve advanced methodologies, including microwave-assisted and ethanol extraction, combined with chromatographic techniques. These approaches enable the isolation of high-purity polysaccharides, phenolics, and fatty acids (FAs). Analytical tools such as NMR, FTIR, and HPLC-MS/MS confirm the presence and quality of these valuable bioactives. Collectively, these findings underscore the potential of *R. okamurae* as a sustainable and eco-friendly resource for diverse industrial applications.

### 3.2. Terpenoids

*R. okamurae* is a rich source of structurally diverse terpenoids, with a particular abundance of biologically active diterpenoids. Chemical investigations of this alga have led to the isolation of several novel compounds exhibiting significant bioactivity.

The major terpenoids identified belong to the diterpenoid class and include almost 40 compounds such as rugukadiol A, rugukamurals A–C, ruguloptones A–F, and okaspatols A–D (**16**–**19**) [6,7,53]. Notably, many of these are secospatane diterpenoids, distinguished by their oxygenated functional groups—rugukamurals and ruguloptones are key representatives of this subclass [6]. Additional diterpenoid subclasses identified in *R. okamurae* include prenylcubebane and prenylkelsoane, exemplified by compounds such as okacubols A and B and okamurol A [7].

**Figure 7:**
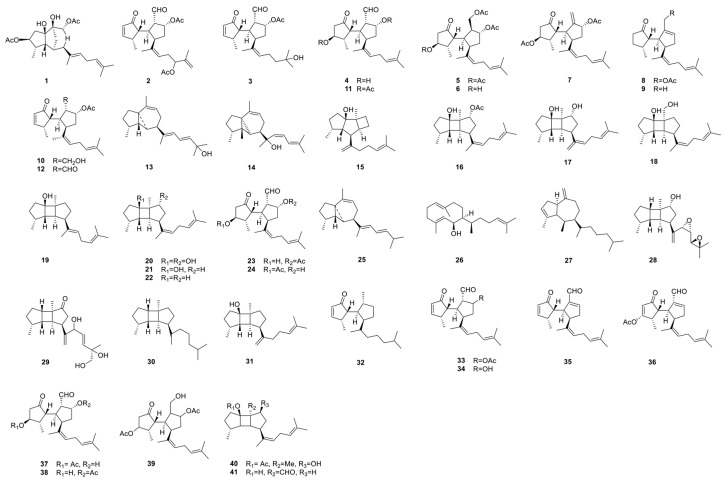
Chemical structures of the diterpenoids isolated from *R. okamurae*.

Several diterpenoids isolated from *R. okamurae* exhibit unique structural features. For example, rugukadiol A possesses a distinctive bridged tricyclic undecane skeleton, representing a novel molecular framework among known diterpenoids [6]. Similarly, okacubols A and B feature a rare kelsoane-type tricyclic nucleus, which is uncommon within this class of compounds [7]. The detailed chemical structures of these diterpenoids are illustrated in Figure 7, and the activities and characteristics of the main types are summarised in Table 3.

In addition to their structural novelty, several diterpenoids from *R. okamurae* demonstrate significant anti-inflammatory activity. Rugukadiol A and secospatane-type diterpenoids such as ruguloptone F have been shown to significantly inhibit nitric oxide (NO) production in lipopolysaccharide (LPS)-stimulated microglia and macrophages [6,7]. These compounds also markedly reduce the expression of key pro-inflammatory mediators, including *Nos2* and the cytokine *Il1b*, in immune cell models [6]. Among the isolated compounds, okaspatol C was particularly noteworthy, as it completely suppressed LPS-induced NO production in both microglial and macrophage assays, highlighting its potential as a highly effective anti-inflammatory agent [7].

In summary, *R. okamurae* is a promising source of structurally diverse and biologically active diterpenoids, with significant potential for therapeutic applications, particularly in the field of inflammation-related disorders.

### 3.3. Phenolic Compounds

#### Phenolic Content

*R. okamurae* has been extensively studied for its phenolic content and associated bioactive properties. Phenolic compounds in this brown alga have been identified and quantified using different extraction and analytical techniques, revealing a diverse and potent antioxidant profile.

The total phenolic content (TPC) of *R. okamurae* varies considerably depending on the extraction method and solvent used. Methanolic extracts have demonstrated relatively high TPC values, reaching up to 17.67 mg gallic acid equivalents per gram (mg GAE/g), along with a total flavonoid content (TFC) of 42.99 mg quercetin equivalents per gram (mg QE/g), as reported by El Madany et al. [54]. In contrast, microwave-assisted extraction yielded a lower TPC of 2.7 mg GAE/g [32], while Cebrián-Lloret et al. [55] reported a total phenol percentage of just 4.5%. Rivero-Pino et al. [52] quantified the polyphenol content as 0.0074 mg of gallic acid per mg of sample.

**Figure 8:**
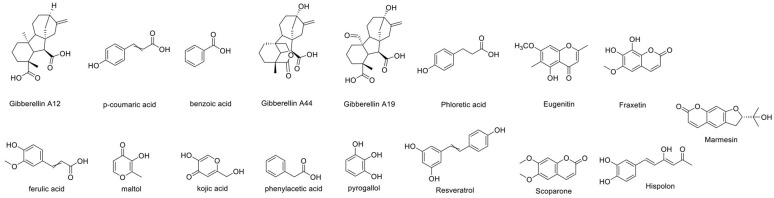
Structures of the main phenolic compounds found in *R. okamurae*.

Among the phenolic compounds identified in *R. okamurae*, gallic acid is the most abundant compound, present at a concentration of 20.7 mg/g [32]. Chlorogenic acid has also been detected. Mass spectrometry analysis has revealed the presence of several additional phenolics—many of which are commonly found in terrestrial plant sources such as olives, berries and tree bark. Key compounds detected (as illustrated in Figure 8), include Gibberellin A12 (265.55 ppb), *p*-coumaric acid (91.87 ppb), benzoic acid (83.28 ppb), Gibberellin A44 (76.57 ppb), Gibberellin A19 (67.60 ppb), and phloretic acid (41.50 ppb).

A range of extraction techniques has been employed to optimise the recovery of phenolic compounds from *R. okamurae*. Ethanol extraction—particularly using 70% ethanol—has proven to be especially effective, yielding up to 310.7 mg GAE/100 g of dry weight [39]. Alternative methods, such as zeolite-assisted milling and microwave-assisted extraction, have also been investigated to enhance efficiency. Notably, ethanol extracts significantly higher amounts of polyphenols compared to water, whereas aqueous extractions tend to yield more reducing sugars. This highlights the importance of selecting appropriate extraction conditions based on the targeted bioactive compounds.

In addition to solvent choice, processing parameters such as drying temperature and milling time can also influence the yield and composition of phenolic extracts. Figueroa et al. [16] and De la Lama-Calvente et al. [56] demonstrated that careful optimisation of these variables can substantially improve both the concentration and diversity of phenolic compounds extracted from *R. okamurae*.

Among brown seaweeds, phlorotannins are regarded as the dominant and most bioactive class of polyphenols, attributed to their unique molecular structure and potent antioxidant properties. In *R. okamurae*, phlorotannins have been extracted at concentrations of 0.35 g/100 g of dry weight [8].

Phenolic compounds are secondary metabolites synthesised via the shikimic acid and phenylpropanoid pathways. They play crucial roles in regulating plant structure, growth, and pigmentation [57]. Moreover, they provide defence against ultraviolet radiation, herbivorous insects, predators, and a wide range of plant pathogens [57,58]. Phenolics derived from natural sources are well-known for their antioxidant, anti-inflammatory, anticancer, and antimicrobial activities. Their antioxidant capacity stems from their redox properties, enabling them to quench singlet oxygen, donate electrons, and transfer hydrogen atoms [57].

In *R. okamurae*, phenolic compounds function as antimicrobial, antifungal, and antioxidant agents, contributing to the alga’s defences by inhibiting microbial invasion [58]. They also reinforce the cell wall and act as chemical signals that can attract or repel neighbouring organisms. The phenolic profile of *R. okamurae* underpins various biological activities essential for both defence mechanisms and physiological regulation, including antioxidant, antimicrobial, anti-inflammatory, and antiproliferative effects [59].

Given this broad spectrum of bioactivities, phenolic compounds from *R. okamurae* hold significant promise as lead compounds in pharmaceutical development. Their demonstrated anti-ageing, anti-inflammatory and anti-proliferative properties are particularly relevant for managing conditions associated with oxidative stress [59]. The strong redox potential of these compounds further supports their potential as preventive and therapeutic agents against chronic diseases driven by oxidative damage [57].

### 3.4. Lipids and Fatty Acids of R. okamurae

Brown seaweeds are notable for their lipid content, which includes a variety of bioactive compounds with potential health-promoting properties. Their lipid profile is diverse, comprising glycoglycerolipids, phospholipids, sphingolipids, and betaine lipids [60,61,62]. The total lipid (TL) content of brown seaweeds typically ranges from 0.13% to 21.3% of dry weight [60,63], although this can vary depending on environmental factors such as depth, water temperature, and salinity [64].

Among the lipid classes, glycoglycerolipids (GLs) are generally predominant and are particularly enriched in polyunsaturated FAs (PUFAs), including eicosapentaenoic acid (EPA, 20:5*n*−3) and arachidonic acid (AA, 20:4*n*−6) [60,61,64]. Phospholipids and sphingolipids also contribute substantially to the lipid fraction, containing bioactive molecules such as ceramide and hexosylceramide [61]. In addition, betaine lipids are present in appreciable amounts, further enhancing the overall nutritional and functional value of these seaweeds [61,62].

#### 3.4.1. Lipids Content

Total lipids are commonly reported in the literature either as lipid extract yield or as total FA content, typically determined using chromatography techniques (Table 4).

#### 3.4.2. Fatty Acid Profiles

The FA profiles of *R. okamurae* as reported by various authors are summarized in Table 5.

The FA profile of *R. okamurae* is predominantly composed of saturated FAs (SFAs), with palmitic acid (16:0) and myristic acid (14:0) representing a significant proportion of the total lipid content. Monounsaturated FAs (MUFAs), particularly oleic acid (OA, 18:1*n*−9), are present in moderate amounts. PUFAs include nutritionally valuable compounds such as ARA and EPA. Most studies report a favourable *n*−6/*n*−3 PUFA ratio, typically below 1—highlighting the nutritional quality of the lipid fraction. However, some variation exists based on factors such as sampling location, season, and extraction methodology. Notably, one study [55] reported a markedly different FA profile, including unusually high levels of 16:4*n*−3 and low EPA, suggesting possible taxonomic misidentification or methodological discrepancies. Overall, the lipid composition of *R. okamurae* supports its potential for nutritional and biotechnological applications.

#### 3.4.3. Lipidic Fraction

**Figure 9:**
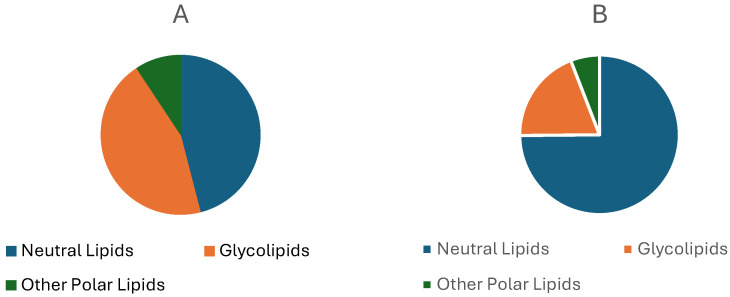
Lipid fractions (mg/g dry wt) of *R. okamurae* collected in 2022 (**A**) and 2023 (**B**), as reported by Córdoba-Granados et al. [65].

**Figure 10:**
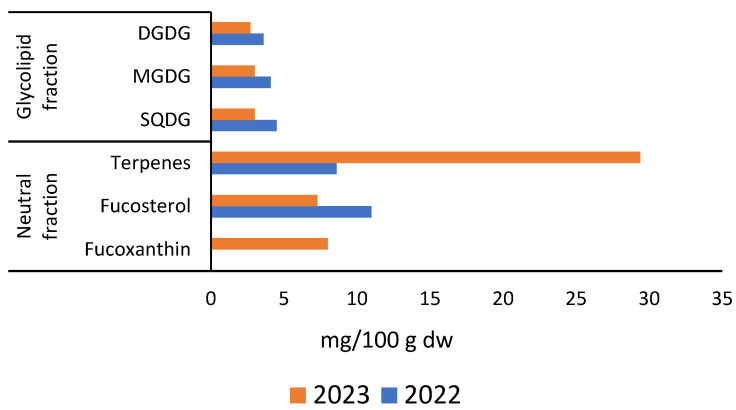
Lipid compounds identification and quantification (mg/g dry weight) of *R. okamurae* collected in 2022 and 2023, as reported by Córdoba-Granados et al. [65]. Legend: SQDG, sulfoquinovosyldiacylglycerol; MGDG, monogalactosyldiacylglycerol; DGDG, digalactosyldiacylglycerol.

The lipid fractions (mg/g dry weight) of *R. okamurae* are presented in Figure 9, while the specific lipid compounds identified are illustrated in Figure 10, as reported by Córdoba-Granados et al. [65].

Córdoba-Granados et al. [65] performed solid-phase extraction on the total lipids of *R. okamurae*, using silica cartridges to separate the lipids into three main classes: neutral lipids, glycolipids, and other polar lipids. These fractions were sequentially eluted with tert-butyl methyl ether, acetone:methanol (9:1, *v*/*v*), and methanol, respectively, and quantified by gravimetric analysis following solvent evaporation. Lipid classes were separated and quantified using high-performance thin-layer chromatography (HPTLC) on silica gel plates, with sample application using a Linomat 5 system. Separation was performed using polar and neutral solvent systems, and densitometric quantification was performed after cupric acetate staining and charring. Phospholipids were identified using the molybdenum blue method.

Significant year-to-year differences were observed in the lipid fractions of *R. okamurae*, including neutral lipids, glycolipids, and other polar lipids (Figure 9). Depending on the fraction, approximately 40–60% of the compounds were identified and quantified. Notably, the neutral fraction showed a marked increase in the 2023 samples (Figure 9A), doubling compared to the previous year. This rise was primarily attributed to an elevated terpenes content (Figure 10). Additionally, fucosterol and the carotenoid pigment fucoxanthin were detected within the neutral fraction, with their concentrations showing significant interannual variation (Figure 10). In contrast, the glycolipid fraction decreased by approximately 50% in 2023 relative to 2022, particularly due to reductions in sulfoquinovosyldiacylglycerides, (SQDG), digalactosyldiacylglycerol (DGDG), and monogalactosyldiacylglycerol (MGDG). The levels of other polar lipids remained relatively stable across both years.

#### 3.4.4. Fatty Acid Profiles of *R. okamurae* as a Chemotaxonomical Tool

The FA composition of *R. okamurae* serves as a valuable chemotaxonomic marker for species identification. This brown alga consistently exhibits a lipid profile dominated by SFAs, particularly PA, alongside moderate levels of MUFAs, such as OA, and PUFAs, notably ARA and EPA. The combined proportion of ARA and EPA generally ranges from 4% to 13% of total FAs, offering a consistent biochemical signature [66].

Moreover, *R. okamurae* typically displays a low *n*−6/*n*−3 PUFA ratio—often below 1— further supporting its biochemical distinctiveness. Overall, FA profiling offers a reliable and informative approach for confirming the identity of *R. okamurae*, particularly when applied alongside morphological and genetic identification methods.

### 3.5. Volatile Compounds

**Figure 11:**
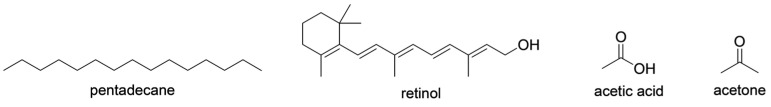
The main individual volatile compounds present in *R. okamurae*.

Rivero-Pino et al. [52] characterised the volatile compound profile of *R. okamurae*, identifying a total of 114 volatile constituents. The most abundant chemical classes were aliphatic hydrocarbons (34.66%), followed by terpenes (27.71%), alcohols (12.33%), and carboxylic acids and ketones (approximately 7%). The dominant individual compounds included pentadecane (29.28%), retinol (6.68%), acetic acid (6.00%), and acetone (4.21%), mirroring profiles commonly reported for other brown algae (Figure 11).

The notable presence of retinol—a form of vitamin A authorised for use in food and supplements—is particularly relevant due to its well-documented health benefits. Retinol holds nutritional and cosmetic value, especially in anti-ageing formulations, owing to its role in collagen synthesis and protection against oxidative stress.

Other naturally occurring volatiles, such as acetone, 2-ethylhexan-1-ol and *trans*, *trans*-2,4-nonadienal, may contribute to the aroma and flavour profile of the alga. However, safety concerns must be addressed: certain compounds, such as thujone, have demonstrated toxicity in animal studies. Therefore, comprehensive safety assessments of the volatile profile are essential before *R. okamurae* can be considered for use in food or feed applications. Future research should also investigate the effects of processing methods on the composition and safety of these volatile compounds.

Although data on the volatile compounds of *R. okamurae* remain limited, the detection of retinol is a particularly noteworthy finding. This highlights the need for more comprehensive metabolomic studies to fully characterise its volatile profile. Such investigations could elucidate the ecological functions of these compounds and unlock new applications in the cosmetic, pharmaceutical, and food industries.

Algae are known to produce a broad spectrum of volatile compounds spanning various chemical families. Terpenes are especially abundant and include compounds such as linalool, geraniol, citronellol, limonene, *α*-pinene, *β*-pinene, 1,8-cineole, and cadinene. Other common volatiles include aromatic compounds such as eugenol and isoeugenol, alongside a range of alcohols, aldehydes, ketones, acids, amines, sulphur-containing compounds, and halogenated compounds [67].

Comparable studies in other plant species have revealed similarly complex volatile profiles. For instance, *Agastache rugosa* produces menthone, pulegone, and estragole [68,69], while *Erechtites valerianaefolia* emits limonene, myrcene, and *trans*-*β*-farnesene [70]. Volatiles from *Combretum* species include palmitic acid, hexahydrofarnesyl acetone, and isophytol [71]. These examples underscore the chemical diversity of natural volatiles and suggest that *R. okamurae* may also possess a unique and potentially valuable volatilome worthy of further exploration.

### 3.6. Peptides

Twenty peptide sequences (<1000 Da) were identified from *R. okamurae* using advanced mass spectrometry combined with in silico bioinformatic analysis by Rivero-Pino et al. [52]. Their physicochemical properties—including hydrophobicity, solubility, and net charge—were predicted using ToxinPred, providing insight into factors relevant to bioavailability, membrane permeability, and stability under physiological conditions.

Further structural analysis with PASTA 2.0 enabled the prediction of aggregation-prone regions, intrinsic disorder, and secondary structure tendencies (e.g., *α*-helix, *β*-strand, and random coils). These features offer valuable information on the peptide’s conformational stability and interaction potential.

Notably, all identified peptides exhibited a 100% probability of intrinsic disorder, indicating a high degree of structural flexibility. This characteristic may enhance their adaptability to various receptor environments, a desirable trait for molecular recognition and therapeutic targeting. Aggregation-prone segments were either absent or confined to short regions (e.g., residues 1–4 or 4–7), indicating a low risk of self-association—an important consideration in drug development. Amphipathicity values ranged from 0.25 to 1.39, reflecting diverse membrane interaction capacities and variable absorption potential.

As summarised in Table 6, the peptides extracted from *R. okamurae*, characterised by LC-MS/MS and analysed through ToxinPred, PASTA 2.0, and molecular docking, exhibit favourable solubility, balanced charge, and low aggregation propensity. Their high conformational flexibility supports strong binding potential, highlighting them as promising candidates for bioactive functions, including anti-inflammatory, antihypertensive, and antidiabetic activities.

## 4. Biological Activity of *R. okamurae*

### 4.1. Antioxidant Activity

Extracts of *R. okamurae* prepared using ethyl acetate, methanol, and chloroform have demonstrated notable antioxidant capacity, with the most potent effects observed in extracts prepared using highly polar solvents such as methanol [54]. This antioxidant potential is further supported by strong free radical inhibition noted in both aqueous and ethanolic extracts [52]. Additionally, these extracts display dose-dependent radical-scavenging activity, underscoring the species’ potent antioxidant properties [32].

Phenolic compounds play a central role in this activity, as confirmed by various assays, including DPPH and ABTS, which consistently show higher antioxidant performance in extracts prepared with polar solvents [32,54]. A summary of phenolic content and associated antioxidant activity is presented in Table 7.

A clear correlation has been established between antioxidant capacity and total phenolic content, indicating that phenolic compounds are key contributors to the free radical-scavenging efficacy of *R. okamurae* extracts [32,54].

Among the bioactive compounds identified in *R. okamurae*, diphlorethohydroxycarmalol—isolated from 70% ethanolic extracts—has emerged as a particularly potent antioxidant [72,73]. Both aqueous and ethanolic extracts of the alga also contain high levels of phenolic compounds and volatile constituents, further contributing to its overall antioxidant potential [52]. The consistently high concentration of phenols and flavonoids across various extract types highlights their central role in the antioxidant activity of *R. okamurae* [32].

Beyond its antioxidant capacity, the nutritional composition of *R. okamurae*—rich in proteins, lipids, carbohydrates, essential nutrients, and trace minerals—positions it as a promising candidate for applications in the food industry [52,54]. Its diverse bioactive profile supports the development of functional foods, nutraceuticals, and biotechnological products, while the antioxidant properties of its extracts suggest potential uses in medicinal, cosmetic, and preservative formulations [32]. In this context, the use of marine invasive species such as *R. okamurae* as skincare ingredients has also been proposed, with the Azorean islands suggested as a case study for biomass valorisation [74]. In comparative studies with other marine algae, *R. okamurae* has consistently demonstrated superior free radical scavenging capacity [75]. These findings are supported by subsequent evaluations that identify the species as one of the most promising sources of antioxidant-rich compounds for functional ingredient development [76].

### 4.2. Anti-Inflammatory Activity

Notable anti-inflammatory effects have been demonstrated by *R. okamurae*, particularly in a mouse model of atopic dermatitis, where its extract significantly reduced ear oedema, mast cell infiltration, and pro-inflammatory cytokine levels [7,72]. These effects are largely attributed to diphlorethohydroxycarmalol, a potent phlorotannin found in the alga.

At a molecular level, *R. okamurae* modulates inflammatory responses by downregulating NF-*κ*B activation and decreasing the production of nitric oxide and reactive oxygen species. It also inhibits upstream signalling molecules involved in inflammation [77,78]. Diphlorethohydroxycarmalol further attenuates inflammation induced by pro-inflammatory cytokines such as TNF-*α* and IFN-*γ*, highlighting its potential as a therapeutic agent for treating inflammatory skin disorders [72].

When compared with other plant-derived agents, *R. okamurae* matches or surpasses them in anti-inflammatory efficacy. For example, polyphenol-rich extracts from *Melaleuca rugulosa* have been shown to inhibit inflammation-associated enzymes and effectively suppress pro-inflammatory mediators [79]. Compounds such as terflavin C and rosmarinic acid from *M. rugulosa* also provide benefits in managing inflammation, oxidative stress, and skin ageing.

Overall, bioactive compounds from *R. okamurae*, particularly diphlorethohydroxycarmalol and various diterpenoids, represent promising leads for the development of novel anti-inflammatory therapies. Alongside other natural sources such as *M. rugulosa*, they hold significant promise for the future development of drugs targeting inflammatory and oxidative stress-related conditions [7,72,79].

### 4.3. Antimicrobial Activity

Seaweeds are known to produce a diverse array of bioactive compounds, including polysaccharides, fatty acids, phlorotannins, pigments, lectins, alkaloids, terpenoids, and halogenated metabolites, which exhibit notable antimicrobial activity [80,81]. Brown seaweeds are particularly rich in sulphated polysaccharides, such as fucoidans, which have demonstrated strong antibacterial effects [82,83]. However, the antimicrobial efficacy of seaweed extracts depends on various factors, including the extraction method, the type of target microorganism, and environmental conditions that affect algal metabolism [80].

In addition to their antibacterial activity, seaweeds also exhibit promising antiviral properties. Fucoidans from brown algae have been reported to inhibit viruses such as HSV-2 and CVB-3 by blocking viral adsorption and entry into host cells [82]. Similarly, carrageenans derived from red algae have shown to selectively inhibit dengue virus serotypes [84], highlighting the broader antiviral potential of algal polysaccharides.

Given its classification as a brown alga, *R. okamurae* is likely to contain similar sulphated polysaccharides and other antimicrobial constituents. Although direct studies are scarce, its biochemical similarity to other brown seaweeds suggests that it may inhibit bacterial and fungal growth through mechanisms such as membrane disruption and enzymatic inhibition. Furthermore, it may interfere with viral adsorption and replication, particularly against pathogens such as HSV-2 and CVB-3.

In summary, while specific data on the antimicrobial and antiviral properties of *R. okamurae* remain limited, evidence from related brown seaweed species indicates strong potential. Further targeted research is needed to confirm and characterise these effects, which could support the development of new therapeutics or preservatives.

### 4.4. Cytotoxic and Anticancer Potential

*R. okamurae* shows great promise in cancer research, largely thanks to its high content of bioactive compounds with antioxidant and anti-inflammatory properties. It is rich in carbohydrates, fats, essential nutrients and polyphenols, including phenolic and volatile compounds such as retinol [32,52]. These constituents contribute to its strong antioxidant capacity, as demonstrated by its significant ability to inhibit free radicals, which may help counteract oxidative stress—a key factor in cancer development.

Ten new diterpenoids, including rugukadiol A and ruguloptones A–F, have been isolated from *R. okamurae* and have demonstrated potent anti-inflammatory activity by inhibiting crucial immune mediators [6]. Such compounds, commonly found in marine organisms, often exhibit cytotoxic properties that may be valuable in anticancer therapies.

While direct studies on the cytotoxic effects of *R. okamurae* are limited, its bioactive profile suggests potential anticancer activity. Similar compounds found in other brown seaweeds have been shown to induce apoptosis in cancer cells, cause cell cycle arrest and reduce oxidative stress—mechanisms typically associated with effective anticancer agents [85,86].

In this regard, brown seaweeds such as *Sargassum* spp. and *Fucus vesiculosus* have exhibited significant anticancer activity, suggesting that *R. okamurae* may hold similar potential, given its comparable bioactive composition [86,87].

### 4.5. Anticoagulant and Immunomodulatory Effects

*R. okamurae* is rich in carbohydrates, lipids, essential nutrients, and polyphenols, including compounds with recognised antioxidant properties [32]. Although its anticoagulant and immunomodulatory effects have not been directly investigated, its bioactive composition suggests potential in both areas. Table 8. Details the anticoagulant and immunomodulatory potential of *R. okamurae*.

Although no specific evidence of anticoagulant activity exists for *R. okamurae* itself, sulfated polysaccharides—such as fucoidans found in related brown algae—have demonstrated significant anticoagulant and antithrombotic properties by prolonging plasma coagulation time [88,89,90]. Given the likelihood that *R. okamurae* contains structurally similar polysaccharides, it may exhibit comparable properties.

Its immunomodulatory potential is supported by its high polyphenol content and antioxidant capacity, both of which are known to influence immune function [32]. Moreover, studies on other fucoidan-rich seaweeds have revealed enhanced immune responses, including increased cytokine production and immune cell activation [89,90,91]. This suggests that *R. okamurae* may also act through similar mechanisms.

**Table 8:** Anticoagulant and Immunomodulatory Potential of *R. okamurae*.

Property	*R. okamurae*	Other Marine Polysaccharides (e.g., Fucoidans)	References
Anticoagulant Activity	Not directly studied	Extends plasma coagulation time; antithrombotic	[88,91]
Immunomodulatory Activity	High polyphenol content; antioxidant activity	Enhances cytokine production; immune cell activation	[32,89,90,91]

## 5. Mechanisms of Action and Bioactivity Correlations

### 5.1. Structure–Activity Relationships

*R. okamurae* contains a variety of bioactive compounds whose structure–activity relationships (SAR) provide valuable insights into their pharmacological potential.

Key components—such as polyphenols and fatty acids, and newly identified diterpenoids—contribute to the alga’s antioxidant and anti-inflammatory activities [6,32,92]. These effects are linked to structural features that influence their interaction with biological targets.

Chemical analyses using spectroscopy and mass spectrometry have characterised its metabolite profile, while bioactivity assays—including molecular docking and anti-inflammatory testing—have confirmed the activity of key peptides and terpenoids [93,94].

Understanding these SARs can inform the development of therapeutic agents and support commercial strategies in areas such as biogas production, composting, bioplastics and pharmaceuticals. Potential applications include anti-inflammatory, antibacterial, and *α*-glucosidase inhibitory activities [93], as well as compost, biostimulants, aquafeed supplements, and biomaterials for the development of eco-friendly products [16].

In summary, *R. okamurae* is a promising source of bioactive molecules with biomedical and biotechnological relevance.

### 5.2. Cellular and Molecular Targets

*R. okamurae* is notable for its rich profile of bioactive compounds with antioxidant, antimicrobial, and therapeutic properties. Studies have demonstrated its ability to inhibit I*κ*B kinase (IKK), a key regulator of inflammatory pathways, as well as its antibacterial activity, which is mediated by disruption of thiamine biosynthesis through the targeting of ThiD kinase [95].

Proteomic analysis has identified 626 unique peptides within the alga, including 21 low-molecular-weight peptides that inhibit angiotensin-converting enzyme (ACE) and dipeptidyl peptidase-IV (DPP-IV), both involved in regulating blood pressure and glucose metabolism [52]. Its potent antioxidant activity is attributed to its high polyphenol content—particularly gallic acid and chlorogenic acid—which combat oxidative stress [32]. Additionally, essential FA such as omega-3, PA, and eicosatetraenoic acid contribute to cellular protection and membrane integrity.

This alga is also a valuable source of minerals, including calcium, potassium, and magnesium, which play key roles in enzyme activity, muscle contraction, and signal transduction. Overall, its bioactive composition suggests therapeutic potential in managing hypertension, diabetes, cardiovascular disorders, and other conditions associated with oxidative stress.

From an ecological perspective, the impact of *R. okamurae* on marine ecosystems is under investigation. A 24-week study on the sea urchin *Paracentrotus lividus* revealed increased consumption and biochemical alterations after prolonged exposure, indicating potential consequences for trophic dynamics and offering insights into the management of invaded environments [17,96].

In conclusion, the diverse nutritional and pharmacological properties of *R. okamurae* make it a promising candidate for developing functional foods and nutraceuticals.

### 5.3. Synergistic and Additive Effects Among Chemical Compounds

Understanding the synergistic and additive effects of the bioactive compounds found in *R. okamurae* provides valuable insight into its biological potential. This invasive brown alga contains a wide variety of bioactive constituents, particularly diterpenoids such as spatanes, secospatanes, prenylcubebanes, and prenylkelsoanes, which have demonstrated potent anti-inflammatory properties by inhibiting nitric oxide production in immune cells [6,7]. Meanwhile, polyphenols such as gallic and chlorogenic acids contribute significantly to its antioxidant capacity [32].

There is evidence to suggest that these compounds may act synergistically, producing biological effects that are greater than the sum of their individual actions. For example, the co-application of diterpenoids has been shown to enhance anti-inflammatory responses, while combinations of polyphenols appear to increase free radical scavenging activity [6,32]. Additive effects have also been observed, particularly among structurally related compounds, as demonstrated in odour threshold studies involving *γ*-lactones [97].

Beyond their pharmacological relevance, these interactions may serve ecological functions. Certain compounds, such as dilkamural, act as herbivore deterrents, and their combined activity likely enhances the species’ chemical defences, contributing to its invasive success [93]. Furthermore, the alga’s efficient nutrient uptake, coupled with metabolic synergy, may support its rapid proliferation in marine ecosystems [98].

In summary, the interplay of bioactive compounds in *R. okamurae* amplifies its anti-inflammatory and antioxidant properties, reinforcing its potential applications in the food, pharmaceutical, and ecological industries. A deeper understanding of these interactions could inform both the exploitation and ecological management of this invasive species.

## 6. Limitations and Outlook of This Review

While this review provides a comprehensive synthesis of the current knowledge on the bioactive compounds of *R. okamurae*, several limitations must be acknowledged. Firstly, a significant proportion of the existing data is fragmented, with inconsistencies in methodologies for extraction, purification, and compound characterisation. Variations in solvent types, analytical platforms, and reporting units across studies hinder direct comparisons, thereby limiting the establishment of robust structure–activity relationships and the prioritisation of compounds for industrial applications.

Secondly, most assessments of biological activity have been confined to in vitro or in silico settings. While these studies offer valuable mechanistic insights, they cannot replace in vivo pharmacological validation. For most compounds—particularly diterpenoids and phlorotannins—key properties such as toxicity, bioavailability, metabolism, and therapeutic index remain insufficiently characterised.

Thirdly, the ecological variability of *R. okamurae*—influenced by habitat, season, and invasion stage—may significantly impact its metabolite profile. However, the use of standardised sampling protocols and comprehensive environmental metadata is rarely documented, thereby impeding reproducibility and ecological modelling.

To advance the field, future research must prioritise the harmonisation of extraction protocols, the development of robust in vivo models, and the investigation of synergistic effects among compound classes. Integrating omics approaches and green bioprocessing technologies will be essential for scaling applications in nutraceuticals, biomedicine, and environmental management. Realising the full bioeconomic potential of this invasive species will require a concerted, multidisciplinary effort.

## 7. Conclusions and Future Perspectives

*R. okamurae* is a chemically rich invasive brown alga whose diverse bioactive compounds, including diterpenoids, phlorotannins, alginate, fatty acids, and peptides, hold significant promise for biotechnological, pharmaceutical, and nutraceutical applications. Its strong antioxidant, anti-inflammatory, antimicrobial, and potential anticancer properties underscore its relevance not only as a subject of scientific interest but also as a candidate for valorisation efforts in response to its ecological spread.

Although substantial progress has been made in characterising its metabolite profile, several critical areas warrant prioritisation in future research. First, extraction optimisation remains essential. Comparative studies across extraction techniques (e.g., ultrasound-assisted, microwave-assisted, and green solvent systems) are needed to enhance yield, selectivity, and sustainability. Establishing standardised extraction protocols will be key to ensuring reproducibility and scalability.

Second, bioactivity-guided fractionation and compound standardisation should be intensified to isolate, identify, and quantify key therapeutically relevant compounds. Structure–activity relationship analyses and mechanistic studies, including molecular docking and in vitro assays, should be complemented by in vivo validation in appropriate disease models.

Third, safety profiling remains a crucial yet underexplored aspect. Although promising bioactivities have been reported, the toxicity, bioavailability, and pharmacokinetics of *R. okamurae*-derived compounds remain largely unknown. Comprehensive toxicological studies and clinical safety assessments are essential to facilitate regulatory approval and foster consumer confidence.

Finally, integrating *R. okamurae* valorisation into ecosystem management strategies presents a unique opportunity to transform an ecological threat into a valuable bioresource. Realising this potential will require multidisciplinary collaborations across phycology, marine ecology, pharmacology, and industrial biotechnology.

## Figures and Tables

**Table 1:** Comparison of Alginate Extraction Methods.

Parameter	Conventional Chemical Extraction [8]	Microwave-Assisted Hydrothermal Extraction [38]
Solvents/Chemicals Used	Formaldehyde, HCl, Na_2_CO_3_, Ethanol, Acetone	Distilled Water or Seawater
Temperature	65 °C (alkaline extraction)	160–180 °C (microwave heating)
Time Required	Several hours (overnight soaking + 5 h extraction)	5–20 min
Pre-treatment	Yes—pigment removal with formaldehyde and acid preconditioning	No pre-treatment needed
Extraction Mechanism	Solubilization via chemical steps	Dielectric heating with pressurised water
Yield (Alginate)	High (purified, sodium alginate)	Up to 3.2% (crude, calcium alginate)
Purification Steps	Ethanol precipitation, acetone wash, freeze-drying	Dialysis and freeze-drying
Advantages	High-purity product; strong gelling (rich in guluronic acid)	Fast, green, low-chemical process; energy efficient
Limitations	Time-consuming; chemical use	Lower alginate yield; needs further purification
Best Use Case	Biomaterials require precise gelation and structural properties	Quick, sustainable extraction for biorefinery or bulk applications

**Table 2:** Comparison Table of Polysaccharides Characteristics of Brown Algae.

Polysaccharide	Structure	Properties	References
Alginate	Linear, blockwise arrangement of *β*-D-mannuronate and *α*-L-guluronate	Gel-forming, used in tissue engineering and drug delivery	[42,44,45]
Fucoidan	Sulfated *α*-L-fucan, alternating (1→3)- and (1→4)-linked *α*-L-fucopyranose	Anticoagulant, antitumor, antiviral, structural variability	[46,47,51]
Laminaran	*β*-D-glucan, (1→3)-linked backbone with (1→6) linkages	Antitumor, inhibits cancer cell colony formation	[46,48]

**Table 3:** Main Terpenoid Compounds isolated from *R. okamurae*.

Compound	Class	Unique Feature	Biological Activity	References
Rugukadiol A (**1**)	Diterpenoid	Bridged tricyclic undecane system	Inhibits NO production, *Nos2*, and *Il1b* expression	[6]
Rugukamurals A–C (**2**–**4**)	Secospatane Diterpenoids	Oxygenated functions	Anti-inflammatory	[6,7]
Ruguloptones A–F (**5**–**10**)	Secospatane Diterpenoids	Oxygenated functions	Anti-inflammatory	[6]
Okacubols A & B (**13**,**14**)	Prenylcubebane Diterpenoids	-	Anti-inflammatory	[7]
Okamurol A (**16**)	Prenylkelsoane Diterpenoid	Kelsoane-type tricyclic nucleus	Anti-inflammatory	[7]
Okaspatols A–D (**16**–**19**)	Spatanes	Oxygenated	Okaspatols A–D (**16**–**19**)	[7]

**Table 4:** Total lipid content in *R. okamurae* as reported by different authors.

Source	Total Lipid Content (% DW)	Methodology	Notes
Rivero-Pino et al. [52]	21.29%	Hexane/isopropanol extract	-
Cebrián-Lloret et al. [55]	17.3%	Folch method	Concerns over taxonomic accuracy
Córdoba-Granados et al. [65]	4.3–10.0%	Solid-phase extraction was performed for total lipid analysis	-
Guil-Guerrero et al. [66]	2.4–6.8 g FA/100 g DW	Total fatty acid content via GC-FID/GC-MS; indirectly reflects total lipids	Seasonal and geographical variation

**Table 5:** Main Fatty Acids of *R. okamurae* (FA% of total FAs) as Reported by Different Authors.

Fatty Acids	Rivero-Pino [52]	Cebrián-Lloret et al. [55]	Córdoba-Granados et al. [65] ^a^	Guil-Guerrero et al. [66] ^b^
Σ SFA	65.6	61.12	37.3–49.6	57.3–71.3
14:0	8.74	15.15	4.3–6.5	9.9–17.8
15:0	0.95	1.48	0.0–0.9	0.9–2.4
16:0	40.03	31.75	24.0–31.5	30.4–40.5
18:0	13.37	3.13	3.1–7.7	2.2–5.0
20:0	0.54	-	2.5–3.8	3.3–6.0
Σ MUFA	13.3	15.26	26.4–29.9	18.8–27.6
15:1*n*−5	0.26	-	-	0.2–4.6
16:1*n*−7	3.14	1.62	1.8–7.1	3.0–8.7
16:1*n*−5			3.5–7.2	
18:1*n*−9	8.43	12.64	12.4–7.5	11.1–15.5
20:1*n*−9	0.62	-	0.0–0.2	0.1–2.2
Σ *n*−6 PUFA	9.0	5.65	6.5–10.6	8.1–15.0
18:2*n*−6 *trans*	0.1	-	-	0.9–2.1
18:2*n*−6 *cis*	2.38	1.68	1.8–2.6	1.2–2.4
20:2*n*−6	0.14	1.58	0.0–0.2	0.8–3.9
20:4*n*−6	5.06	2.18	1.97–5.9	3.0–6.7
Σ *n*−3 PUFA	12.1	17.98	8.2–11.0	1.8–6.2
16:4*n*−3	-	7.82	-	-
18:3*n*−3	3.41	4.09	1.1–2.1	0.6–2.5
18:4*n*−3	-	3.17	1.0–2.2	0.0–0.7
20:5*n*−3	7.75	0.70	1.6–2.3	0.8–3.2
22:5*n*−3	0.50	1.30	0.3–2.9	-
22:6*n*−3	0.24	-	0.0–0.4	0.0–1.6
Σ PUFA	21.1	23.62	15.1–18.8	13.3–20.4
*n*−6/*n*−3	0.74	0.31	0.79–0.96	2.0–4.5
ARA + EPA	12.85	2.88	3.6–8.2	4.1–9.3

^a^ Variability due to polarity of the extracting solvent and year of collecting; ^b^ Variability due to location of the collecting areas and season.

**Table 6:** Best-Performing Peptides (Bioactivity Potential) as reported by Rivero-Pino et al. [52].

Peptide	Hydrophobicity	Charge	Amphipathicity	Self-Agg	Disorder (%)	Helix (%)	Bioactivity Potential
VGDIARIY	0.01	0.00	0.27	5–9	100	22.22	High solubility, coil–*β* mix, balanced traits
ETGIKVVDL	−0.01	−1.00	0.55	4–7	100	33.33	Highest β-strand (66.67%), soluble, possible structural bioactivity
APILPVVGK	0.17	1.00	0.41	3–7	100	–	Amphipathic, positively charged (ACE/DPP-IV relevance)
TVDAAGKVA	−0.11	1.00	0.55	4–7	100	–	Neutral hydrophobicity, amphipathic, good for receptor binding
VLVGGSTRIP	0.04	1.00	0.25	1–4	100	–	Slightly hydrophobic, basic, low aggregation– stable ligand candidate

**Table 7:** Summary of Phenolic Compounds and Antioxidant Activity.

Extraction Method	Solvent	TPC	Key Phenolic Compounds	Antioxidant Activity	References
Microwave-Assisted	Ethanol	2.7 mg GAE/g	Gallic acid, Chlorogenic acid	Elevated (DPPH, ABTS)	[32,39,54]
Solvent Extraction	Methanol	17.67 mg GAE/g	Not specified	Elevated (DPPH, ABTS)	[32,39,54]
Ethanol Extraction	70% Ethanol	310.7 mg GAE/100 g	Not specified	Elevated (FRAP, ABTS)	[32,39,54]

## Data Availability

No new data were created or analysed in this study. Data sharing is not applicable to this article.
